# Critical Role of TFEB-Mediated Lysosomal Biogenesis in Alcohol-Induced Pancreatitis in Mice and Humans

**DOI:** 10.1016/j.jcmgh.2020.01.008

**Published:** 2020-01-25

**Authors:** Shaogui Wang, Hong-Min Ni, Xiaojuan Chao, Xiaowen Ma, Thomas Kolodecik, Robert De Lisle, Andrew Ballabio, Pal Pacher, Wen-Xing Ding

**Affiliations:** 1 Department of Pharmacology, Toxicology and Therapeutics, University of Kansas Medical Center, Kansas City, Kansas; 2 Digestive Diseases Section, Department of Internal Medicine, Yale School of Medicine, New Haven, Connecticut; 3 West Haven VA Medical Center, VA Connecticut Health System, West Haven, Connecticut; 4 Department of Anatomy and Cell Biology, University of Kansas Medical Center, Kansas City, Kansas; 5 Telethon Institute of Genetics and Medicine, Telethon Institute of Genetics and Medicine, Pozzuoli, Italy; 6 Medical Genetics, Department of Translational Medicine, Federico II University, Naples, Italy; 7 Department of Molecular and Human Genetics, Baylor College of Medicine, Houston, Texas; 8 Jan and Dan Duncan Neurological Research Institute, Texas Children’s Hospital, Houston, Texas; 9 Laboratory of Cardiovascular Physiology and Tissue Injury, National Institute on Alcohol Abuse and Alcoholism, National Institutes of Health, Bethesda, Maryland

**Keywords:** Autophagy, Lysosome, Inflammation, Tissue Injury, Zymogen, ADM, acinar-to-ducal metaplasia, Ad, adenovirus, Atg, autophagy-related, CK19, cytokeratin 19, CLEAR, coordinated lysosomal expression and regulation, EM, electron microscopy, ER, endoplasmic reticulum, GFP, green fluorescent protein, KO, knockout, LC3, microtubule-associated protein light chain 3, MAPK1/ERK2, mitogen-activated protein kinase 1, mTOR, mechanistic target of rapamycin kinase, qPCR, quantitative polymerase chain reaction, TAP, trypsinogen activation peptide, TFEB, transcriptional factor EB, WT, wild-type, ZG, zymogen granule

## Abstract

**Background & Aims:**

Alcohol abuse is the major cause of experimental and human pancreatitis but the molecular mechanisms remain largely unknown. We investigated the role of transcription factor EB (TFEB), a master regulator of lysosomal biogenesis, in the pathogenesis of alcoholic pancreatitis.

**Methods:**

Using a chronic plus acute alcohol binge (referred to as Gao-binge) mouse model, we analyzed pancreas injury, autophagic flux, zymogen granule removal, TFEB nuclear translocation and lysosomal biogenesis in GFP-LC3 transgenic mice, acinar cell-specific Atg5 knockout (KO) and TFEB KO mice as well as their matched wild type mice.

**Results:**

We found that Gao-binge alcohol induced typical features of pancreatitis in mice with increased serum amylase and lipase activities, pancreatic edema, infiltration of inflammatory cells, accumulation of zymogen granules (ZGs) and expression of inflammatory cytokines. While Gao-binge alcohol increased the number of autophagosomes, it also concurrently inhibited TFEB nuclear translocation and TFEB-mediated lysosomal biogenesis resulting in insufficient autophagy. Acinar cell-specific Atg5 KO and acinar cell-specific TFEB KO mice developed severe inflammatory and fibrotic pancreatitis in both Gao-binge alcohol and control diet-fed mice. In contrast, TFEB overexpression inhibited alcohol-induced pancreatic edema, accumulation of zymogen granules and serum amylase and lipase activities. In line with our findings in mice, decreased LAMP1 and TFEB nuclear staining were also observed in human alcoholic pancreatitis tissues.

**Conclusions:**

our results indicate that TFEB plays a critical role in maintaining pancreatic acinar cell homeostasis. Impairment of TFEB-mediated lysosomal biogenesis by alcohol may lead to insufficient autophagy and promote alcohol-induced pancreatitis.

## Graphical abstract

**Figure:**
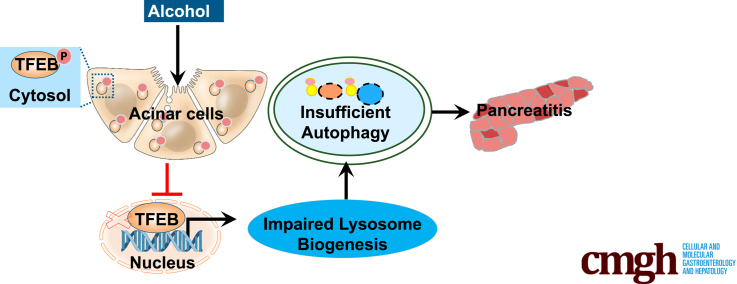

**Summary**

Chronic plus binge alcohol impaired transcription factor EB–mediated lysosomal biogenesis in the mouse pancreas, resulting in insufficient autophagy and pancreatitis. Genetic deletion or overexpression of transcription factor EB in the mouse pancreas exacerbated or protected against alcohol-induced pancreatic damage.

Alcohol abuse has long been considered as a major risk factor for both acute and chronic pancreatitis, but the exact molecular mechanisms in the pathogenesis of pancreatitis are still obscure.^1^ It has been reported that chronic alcohol consumption increases digestive enzyme contents in pancreas by increasing transcription of digestive enzymes^2^ and impairing secretion of zymogen granules (ZGs).^3^ Moreover, alcohol increases the fragility of pancreatic ZGs,^4^ which leads to increased risk of intra-acinar activation of the digestive enzymes. Recent evidence suggests that intracellular activation of digestive zymogens is the initial step of pancreatitis, and selective removal of fragile ZGs can prevent pancreatitis through autophagy.^5^^,^^6^ Autophagy is a cellular catabolic process that requires autophagy-related (Atg) protein-mediated autophagosome formation to transport the cargoes for lysosomal degradation. The direct link between pancreatitis and autophagy-lysosomal pathway was illustrated by deletion of several essential Atg and lysosomal genes, which caused spontaneous pancreatitis.^7^, ^8^, ^9^, ^10^ Moreover, decreased pancreatic LAMP1/2 and lysosomal dysfunction have also been reported in human and mouse alcoholic pancreatitis.^8^^,^^11^ Nevertheless, whether a transcriptional program that governs the autophagy-lysosomal process would play a role in alcoholic pancreatitis remains poorly defined. Mounting evidence suggests that alcohol causes organelle damage including mitochondria, endoplasmic reticulum (ER), and ZGs.^4^^,^^12^^,^^13^ ZGs are specialized storage organelles containing inactive digestive enzymes (ie, trypsinogen) in pancreatic acinar cells. Intrapancreatic trypsinogen activation is considered a key initiating event of pancreatitis that can lead to acinar cell necrosis.^14^ Therefore, maintaining sufficient autophagy degradative capacity is critical for the removal of fragile and damaged ZGs to prevent the pathogenesis of pancreatitis. As lysosome acts at the last stage of autophagy by degrading damaged organelles delivered by autophagosomes, keeping robust lysosomal biogenesis is of great importance to provide a sufficient number of healthy lysosomes to meet the demand for autophagic degradation.

Transcription factor EB (TFEB), which belongs to the microphthalmia family of basic helix-loop-helix-zipper (bHLH-Zip) transcription factors (MiT family),^15^ is a master transcription regulator of lysosomal biogenesis.^16^ Promoter analysis shows that lysosomal genes share a 10-base E-box–like sequence called coordinated lysosomal expression and regulation (CLEAR) motif.^17^ TFEB can directly bind to CLEAR motif and promote the expression of the entire CLEAR network.^18^ Therefore, by modulating the process of lysosomal biogenesis, TFEB coordinates an efficient transcriptional program to control cellular degradation and facilitate intracellular clearance. TFEB is mainly regulated post-translation via phosphorylation. Mechanistic target of rapamycin kinase (mTOR) and the mitogen-activated protein kinase 1/ERK2 (MAPK1) phosphorylate TFEB at Ser142 and Ser211 result in TFEB cytosolic retention, which inactivates TFEB transcription activity.^19^ TFEB has been shown to execute various functions in different tissues. In osteoclasts, TFEB regulates bone resorption through RANKL-PKCβ signaling cascade, and osteoclast-specific deletion of TFEB causes decreased lysosomal gene expression and increased bone mass.^20^ TFEB also modulates lipid catabolism, depletion of TFEB in the liver,^21^^,^^22^ or loss-of-function mutation HLH-30 (TFEB orthologue) in *Caenorhabditis elegans*,^23^ resulting in fat accumulation and impaired lipophagy. We recently reported that TFEB was impaired in caerulein-induced experimental pancreatitis in mice. Mechanistically, caerulein increased mTOR activation and enhanced proteasome activity resulting in TFEB degradation in mouse pancreas.^10^ While caerulein is widely used in the animal experimental pancreatitis model, a super high nonphysiological dose of caerulein is required to trigger pancreatitis in mice and the physiological relevance of this model to human disease is unclear and questionable. The chronic plus acute alcohol binge model (referred to as Gao-binge model) has been successfully used to reproduce steatosis, cell death, and inflammation, which are early pathologic changes of human alcoholic liver disease.^24^ The molecular mechanisms of how alcohol induces pancreatitis are still largely unknown due to the lack of suitable alcohol experimental mouse models. In the present study, we found that protein levels of TFEB decreased in both human and mouse alcoholic pancreas tissues. Impaired TFEB-mediated lysosomal biogenesis induced by alcohol caused insufficient autophagy which contributed to pancreatic injury. Genetic ablation of Atg5 or TFEB specifically in mouse pancreatic acinar cells induced fibrotic pancreatitis in mice fed with either Gao-binge alcohol or liquid control diet. In contrast, overexpression of TFEB ameliorated alcohol-induced ZG accumulation and pancreas injury. Taken together, our data suggest that TFEB-mediated lysosomal biogenesis plays a critical role in the pathogenesis of alcoholic pancreatitis.

## Results

### Alcohol Increases Autophagic Flux in Pancreas and Induces Acute Pancreatitis in Mice

**Figure 1:**
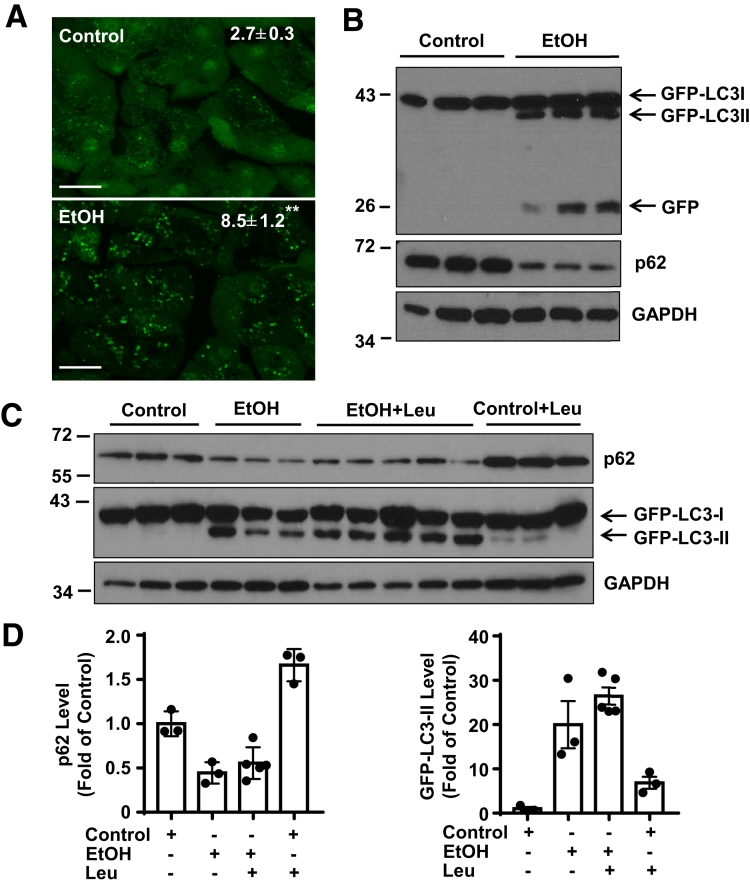
**Alcohol increases autophagic flux in mouse pancreas.** Male GFP-LC3 transgenic mice were administrated with Gao-binge alcohol model. For some mice, on the last day of feeding, 1 dose of leupeptin (40 mg/kg, intraperitoneal) was given to the mice immediately after the gavage of either ethanol or maltose. Mice were sacrificed 8 hours later after the gavage. (*A*) Representative fluorescence microscopy images of cryosections of pancreas are shown. Scale bar = 20 μm. GFP-LC3 puncta per cell in each group were quantified (n = 3). Data are mean ± SE. **P < .01 by Student’s *t* test. More than 30 cells were counted in each mouse. (*B*) Immunoblotting analysis using total lysates from pancreatic tissues after administration of alcohol. (*C*) Total pancreatic lysates were subjected to immunoblotting analysis after alcohol and leupeptin treatments. (*D*) Densitometry analysis of panel *C*. Data are mean ± SE (n = 3–5). Leu, leupeptin.

**Figure 2:**
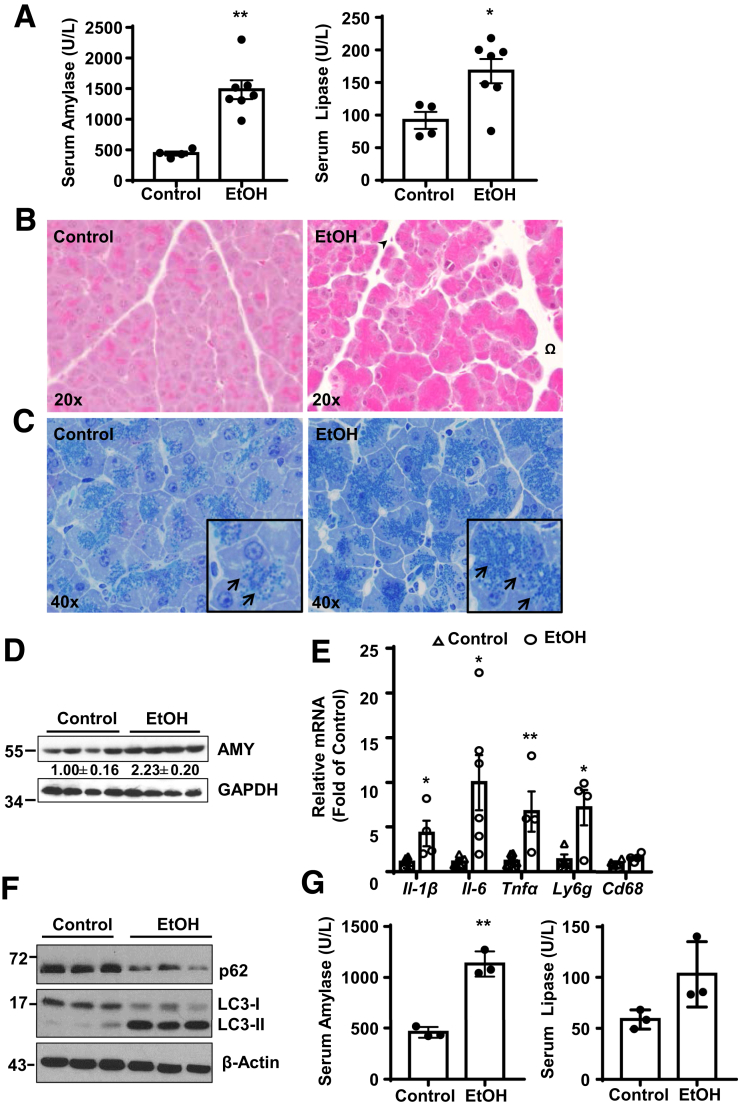
**Alcohol induces edematous pancreatitis in mice.** (*A*) GFP-LC3 transgenic mice were treated with Gao-binge alcohol model. Serum amylase and lipase levels were measured. Data shown are mean ± SE (n = 4–7). *P < .05; **P < .01 by Student’s *t* test. (*B*) Representative hematoxylin and eosin staining images of the GFP-LC3 mouse pancreas from alcohol administration. Omega shows edema and arrow head denotes infiltrated inflammatory cells. (*C*) Representative Toluidine blue staining images from control and alcohol-fed C57Bl/6J mouse pancreas. Arrows denote ZGs. (*D*) Total pancreatic lysates from C57Bl/6J mice were subjected to Western blot analysis. (*E*) Pancreatic messenger RNA (mRNA) were extracted followed by qPCR. Data are mean ± SE (n = 4–7). *P < .05, **P < .01; Student’s *t*-test analysis. (*F*) Immunoblotting analysis of total lysates from C57BI/6J mouse pancreatic tissues. (*G*) Serum amylase and lipase activities were measured from male C57BI/6J mice treated with Gao-binge alcohol. Data shown are mean ± SE (n = 3). **P < .01; Student’s *t*-test analysis.

To determine autophagic flux in mouse pancreas after acute-on-chronic alcohol (referred to as Gao-binge alcohol), green fluorescent protein (GFP)-LC3 transgenic mice were treated with Gao-binge alcohol model. As shown in Figure 1*A*, GFP-LC3 displays a diffuse pattern in the cytosol of acinar cells in control mice. Following Gao-binge alcohol feeding, the number of GFP-LC3 puncta increased due to their targeting to autophagosomal membranes. We further found that alcohol feeding increased GFP-LC3-II and free GFP levels by Western blot analysis, the latter is generated from the degradation of GFP-LC3-II in the autolysosomes.^25^ The levels of p62, a substrate protein of autophagy, markedly decreased after ethanol (Figure 1*B*). Moreover, alcohol feeding further increased the levels of GFP-LC3-II in the presence of leupeptin (a lysosomal inhibitor) compared with either ethanol alone or leupeptin treatment alone (Figure 1*C*, *D*). Based on the guidelines of autophagy, these data suggest that Gao-binge alcohol increased autophagic flux in mouse pancreas.^26^ We found that Gao-binge alcohol-fed GFP-LC3 mice had 2-fold higher serum amylase and lipase activities than mice that were pair-fed with the control diet (Figure 2*A*). Histological analysis showed increased pancreatic edema and inflammatory cell infiltration in alcohol-fed mice (Figure 2*B*). Alcohol also increased the number of ZGs revealed by Toluidine blue staining and levels of amylase by Western blot analysis in mouse acinar cells (Figure 2*C*, *D*). Mice fed with alcohol also had increased expression of inflammatory cytokine genes in pancreas (Figure 2*E*). To rule out the possibility that alcohol-induced pancreatic changes were not due to the GFP-LC3 transgenic mice, we treated the wild type C57Bl/6J mice with Gao-binge alcohol. Similar to GFP-LC3 transgenic mice, Gao-binge alcohol feeding increased levels LC3-II but decreased p62 in mouse pancreas as well as increased serum levels of amylase and lipase (Figure 2*F*, *G*). Taken together, these data indicate that Gao-binge alcohol increased autophagic flux but induced acute pancreatitis in mice.

### Autophagy May Help to Remove Damaged ZGs Induced by Alcohol

**Figure 3:**
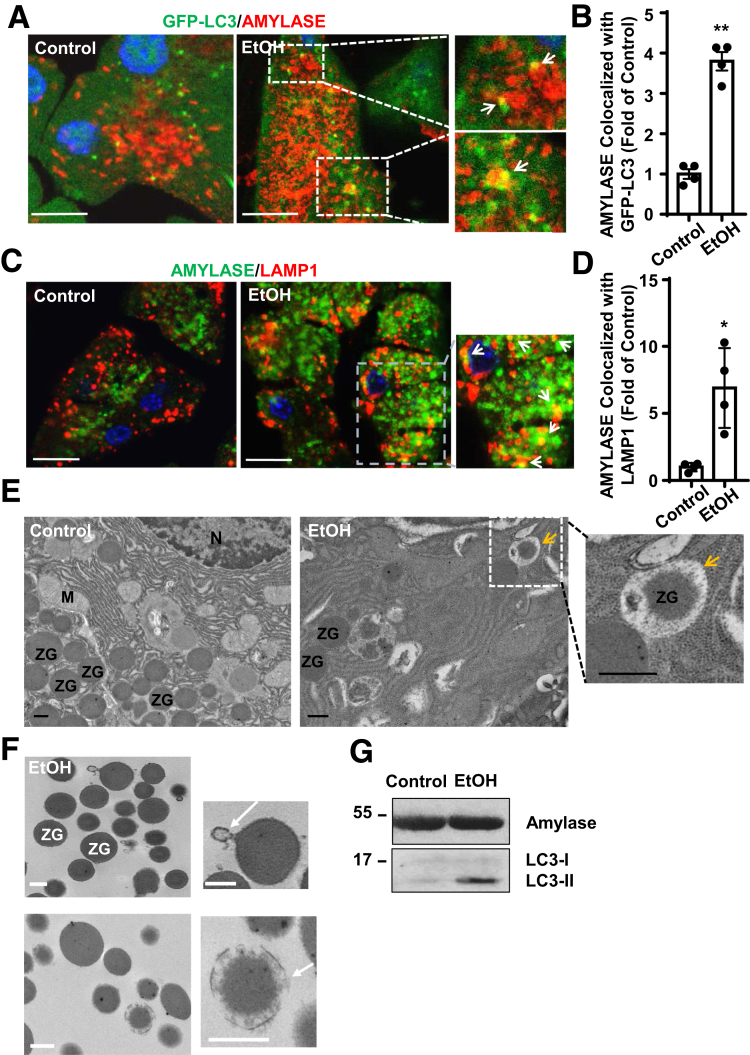
**Alcohol increases colocalization of ZGs with autophagic vacuoles.** Male GFP-LC3 transgenic or WT C57Bl/6J mice were administrated with Gao-binge alcohol model. (*A*) Immunofluorescence staining of AMYLASE (red) was performed using cryosections of GFP-LC3 mouse pancreas followed by confocal microscopy. Arrows denote the colocalization of GFP-LC3 (green) and AMYLASE (red). Scale bar = 40 μm. (*B*) The numbers of GFP-LC3 puncta that are colocalized with AMYLASE were quantified. Data are mean ± SE (n = 4). More than 50 cells were counted in each mouse. **P < .01; Student’s *t* test. (*C*) Immunofluorescence staining of LAMP1 (red) and AMYLASE (green) was performed in cryosections of WT C57Bl/6J mouse pancreas followed by confocal microscopy. Arrows indicate the colocalization of LAMP1 and AMYLASE. Scale bar = 40 μm. (*D*) The numbers of LAMP1 puncta that are colocalized with AMYLASE were quantified. Data are mean ± SE (n = 4). More than 50 cells were counted in each mouse. **P < .01; Student’s *t* test. (*E*) Representative EM images from control and alcohol-fed mice are shown. Arrows denote enwrapped ZGs within autophagic vacuole (AVs). Scale bar = 500 nm. (*F*) Pancreatic ZGs were isolated and purified followed by EM studies and (*G*) immunoblot analysis. Scale bar = 500 nm. M, mitochondria; N, nucleus;

As alcohol consumption has been shown to increase fragility of ZGs in the rat pancreas,^4^^,^^27^ we next questioned if autophagy could remove alcohol-induced damaged ZGs. We found that Gao-binge alcohol feeding increased the colocalization of ZGs with GFP-LC3 puncta (Figure 3*A*, *B*) and LAMP1-positive lysosomes (Figure 3*C*, *D*). Autophagic vacuoles enveloping ZGs (Figure 3*E*, arrows) were also readily detected in acinar cells of alcohol-fed mice**,** but not in acinar cells of control mice. Moreover, some isolated ZGs from alcohol-fed mice were associated with some membrane vesicles or enveloped by autophagosome-like structures (Figure 3*F*, arrows). Western blot analysis also showed increased LC3-II levels in these isolated ZGs in alcohol-fed mice compared with control diet fed mice (Figure 3*G*). These data support the notion that autophagy may help to remove damaged ZGs induced by alcohol.

### Alcohol Induces Insufficient Autophagy in the Mouse Pancreas

**Figure 4:**
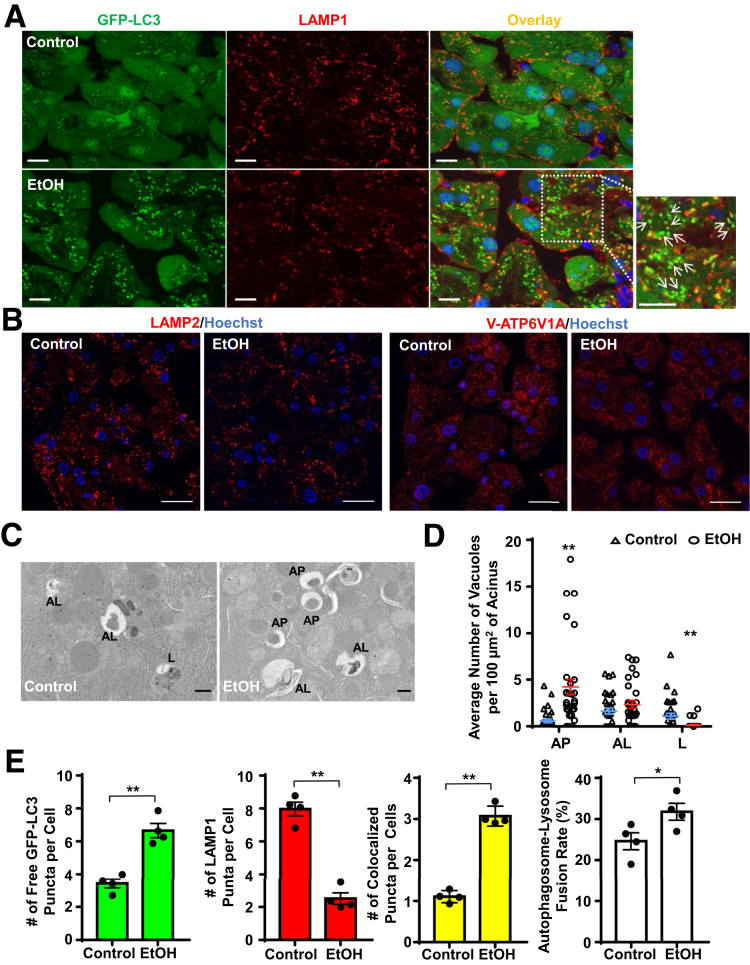
**Alcohol increases the accumulation of autophagosomes in mouse pancreas.** Male GFP-LC3 transgenic or WT C57Bl/6J mice were treated with the Gao-binge alcohol model. (*A*) Cryo-sections of GFP-LC3 mouse pancreas were subjected to immunostaining for LAMP1 followed by confocal microscopy. Representative images of GFP-LC3 and immunostaining of LAMP1 as well as the overlay images are shown. Arrows denote the green-only GFP-LC3 puncta. Boxed areas were enlarged and showed in the lower panel. Scale bar = 10 μm. (*B*) Immunostaining for LAMP2 and ATP6V1A was performed using GFP-LC3 mouse pancreatic cryosections. Scale bar = 20 μm. (*C*) Representative EM images of the WT C57Bl/6J mouse pancreas treated with the Gao-binge alcohol and control diets. (*D*) Quantification of autophagic vacuoles from panel *C*. More than 28 fields from each group were counted for the number of autophagic vacuoles from 2 mice. Scale bar = 500 nm. (*E*) Quantification of numbers of GFP-LC3 only puncta, LAMP1 only puncta, overlap puncta per cell and autophagosome-lysosome fusion ratio from panel *A*. Data are mean ± SE (n = 4). More than 50 cells were counted in each mouse. *P < .05; **P < .01; Student’s *t* test. AP, autophagosome; AL, autolysosome; L, lysosome.

While Gao-binge alcohol increased autophagic flux in the mouse pancreas, these mice still developed pancreatic damage with features of pancreatitis. We recently demonstrated that Gao-binge alcohol impaired TFEB-mediated lysosomal biogenesis in mouse livers, resulting in insufficient autophagy.^22^^,^^28^ We next wondered whether Gao-binge alcohol would also impair lysosomal biogenesis in the pancreas. Indeed, we found that alcohol feeding caused ∼40% reduction of lysosome numbers in acinar cells as judged by LAMP1-positive as well as LAMP2- and V-ATP6V1A–positive vesicles or puncta (labeled as red in Figure 4*A*, *B*). The decreased lysosome numbers in mouse acinar cells after alcohol feeding was further confirmed by the quantification of numbers of autophagosomes, autolysosomes and lysosomes from electron microscopy (EM) studies (Figure 4*C*, *D*). We found that the number of green-only GFP-LC3 puncta (that are not colocalized with LAMP1) was significantly higher in the alcohol-fed mouse pancreas than in the pancreas of control mice. Notably, the net number of GFP-LC3 puncta that colocalized with LAMP1-positive puncta and even the rate of GFP-LC3 puncta fuse with LAMP1-positive puncta all increased in alcohol-fed mouse acinar cells compared with control diet–fed mice (Figure 4*A*, *E*). These data suggest that there are increased numbers of autophagosomes in alcohol-fed mouse acini, which may lead to increased autolysosomes resulting in overall increased autophagic flux (Figure 1). However, autophagic flux in alcohol-fed mouse acinar cells was compromised due to the lack of a sufficient number of lysosomes to meet the need of fusion with increased autophagosomes, which halts the autophagy from reaching the maximal degradation capacity after alcohol feeding (termed insufficient autophagy).^28^

### Alcohol Decreases Nuclear TFEB and TFEB-Mediated Lysosome Biogenesis in Mouse Pancreatic Acinar Cells

**Figure 5:**
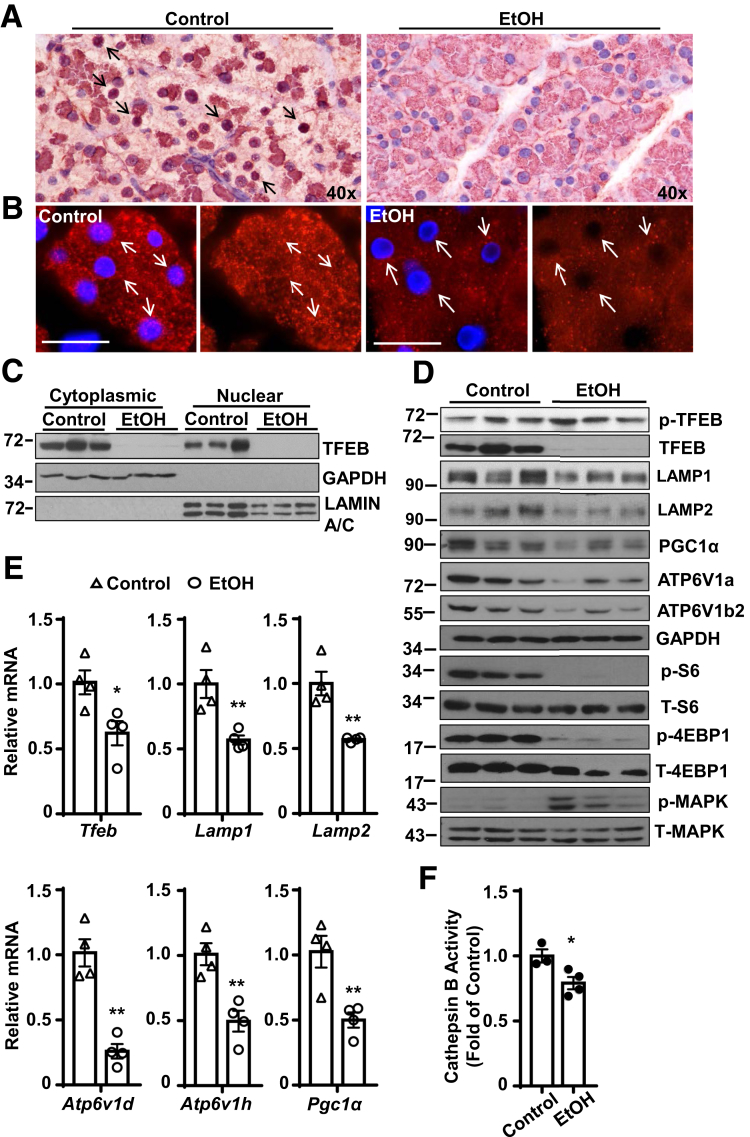
**Alcohol decreases TFEB expression in mouse pancreas.** WT C57Bl/6J mice were administrated with the Gao-binge alcohol model. (*A*) Immunohistochemistry staining for TFEB using paraffin-embedded pancreatic tissues from alcohol treatment. Arrows show nuclear staining of TFEB. (*B*) Immunofluorescence analysis of TFEB staining using cryopancreatic tissues from alcohol treatment. Nuclei were stained with Hoechst33342. Arrows denote nuclear staining of TFEB. Scale bar = 20 μm. (*C*) Immunoblotting analysis using cytoplasmic and nuclear fractions from the mouse pancreas that were administrated with alcohol (*n =* 3). (*D*) Immunoblotting analysis using total lysates from pancreatic tissues after administration of alcohol. (*E*) Pancreatic RNA was extracted and used for qPCR. Results were normalized to 18s and expressed as fold change compared with control group. Data shown are mean ± SE (*n* = 4). *P < .05; **P < .01; Student’s *t*-test analysis. (*F*) Cathepsin B activity was measured from mouse pancreatic tissues. Data are mean ± SE (n = 3-4). **P < .01; Student’s *t*-test analysis.

**Figure 6:**
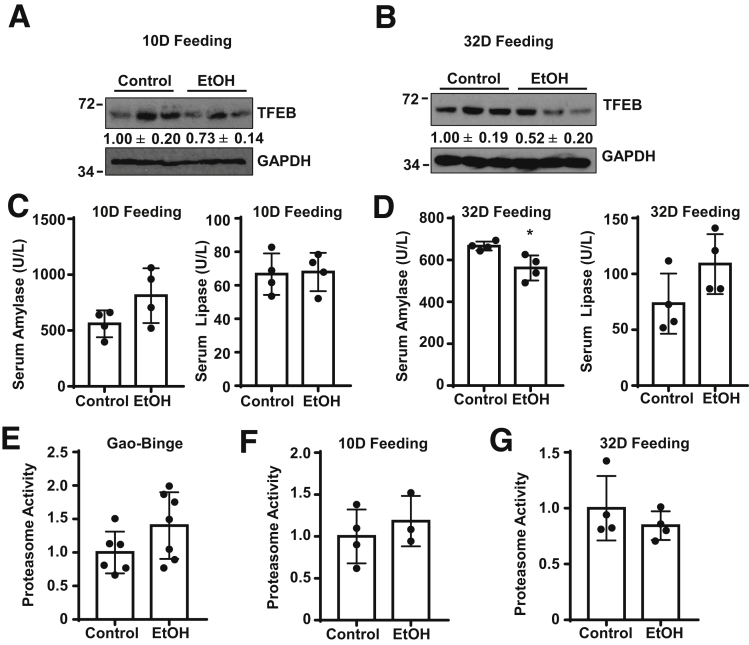
**Ten days or 32 days alcohol feeding without alcohol binge slightly decreases pancreatic TFEB protein in mice.** Male WT C57Bl/6J mice were fed with the Lieber-Decarli liquid ethanol diet for 10 days (10D) or 32 days (32D). (*A*, *B*) Immunoblotting analysis of total lysates from pancreatic tissues. (*C*, *D*) Serum amylase and lipase activities were measured. Data shown are mean ± SE (n = 4). Proteasome activities were measured after (*E*) Gao-Binge alcohol feeding, (*F*) 10D alcohol feeding, and (*G*) chronic 32D alcohol feeding. Data shown are mean ± SE (n = 3–7).

Both immunohistochemical and immunofluorescence staining of TFEB revealed decreased levels of nuclear TFEB in ethanol-treated mouse pancreatic acinar cells (Figure 5*A*, *B*). Consistent with the immunostaining data, Western blot analysis of cellular fractionations from the pancreas also revealed markedly decreased nuclear TFEB protein levels by alcohol (Figure 5*C*). The total levels of TFEB and its target proteins including PGC1α and lysosomal proteins VATP6V1a and VATP6V1b2 as well as in LAMP1 and LAMP2 also marked decreases in the alcohol-fed mouse pancreas (Figure 5*D*). The levels of phosphorylated TFEB slightly increased in the alcohol-fed mouse pancreas. As TFEB can be phosphorylated and inactivated by mTOR or MAPK1,^19^ we next determined whether mTOR or MAPK1 would be associated with decreased pancreatic TFEB by alcohol. We found that alcohol decreased the levels of p-S6 and p-4EBP1, 2 substrates of mTOR, suggesting that alcohol inhibits mTOR in the pancreas and the inactivation of TFEB is less likely mediated by mTOR. In contrast, we found that the levels of p-MAPK markedly increased in the alcohol-fed mouse pancreas, suggesting that MAPK activation may be associated with decreased TFEB by alcohol (Figure 5*D*). In addition to the changes of TFEB proteins and nuclear translocation, we also found that the messenger RNA levels of several TFEB target genes, including *Tfeb*, *Lamp1*, *Lamp2*, *Vatp6v1d*, *Vatp6v1h*, and *Pgc1α* significantly decreased in alcohol-fed mouse pancreas (Figure 5*E*). Moreover, alcohol-fed mice also had decreased cathepsin B activities in mouse pancreas compared with control diet–fed mice (Figure 5*F*). Interestingly, mice fed with a Lieber-DeCarli alcohol diet for 10 days or 32 days without acute binge with alcohol only caused a mild decrease of the pancreatic levels of TFEB (Figure 6*A*, *B*). Mice fed with alcohol for 10 days had slightly increased serum levels of amylase but not lipase (Figure 6*C*). Mice fed with alcohol for 32 days did not affect the serum levels of amylase and lipase (Figure 6*D*). These data suggest that decreased pancreatic TFEB is relatively more specific to the Gao-binge alcohol model. Because TFEB can be degraded by proteasome,^10^ we also determined the pancreatic proteasome activities in these different alcohol mouse models. We found Gao-binge alcohol and 10 days’ feeding with the alcohol diet increased pancreatic proteasome activities approximately 40% or 20%. In contrast, mice fed with the alcohol diet for 32 days showed slightly decreased pancreatic proteasome activities (Figure 6*E–G*). Collectively, these data indicate that TFEB-mediated lysosomal biogenesis is impaired in alcohol-induced pancreatitis in mice.

### Acinar Cell–Specific TFEB Knockout Mice Develop Severe Pancreatitis With the Lieber-DeCarli Liquid Diet Regardless of Ethanol

**Figure 7:**
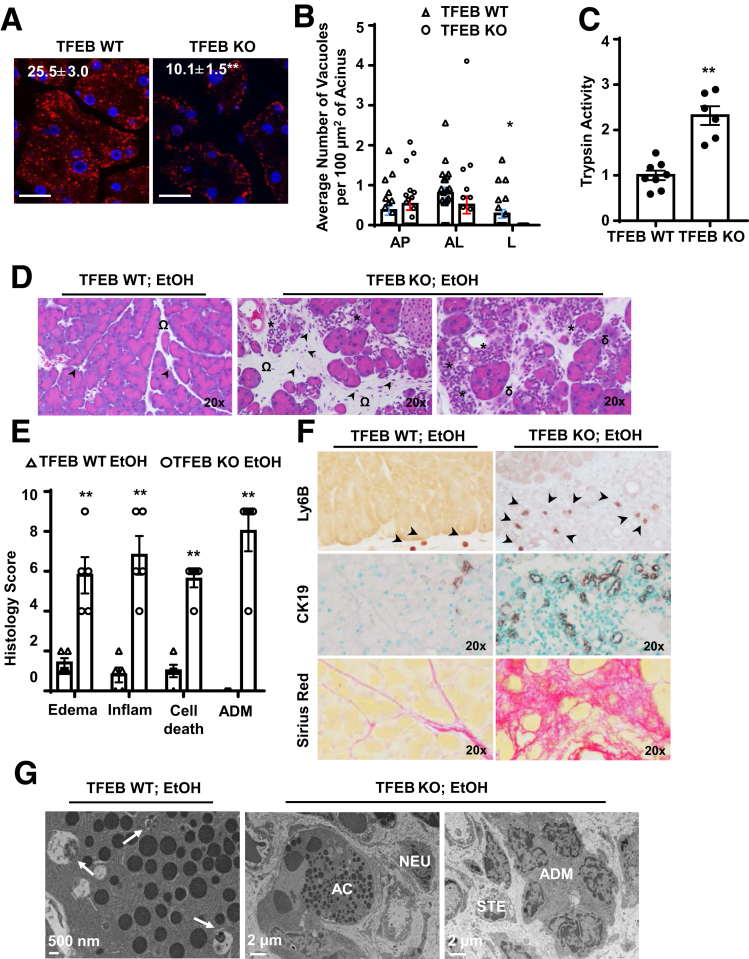
**Acinar cell (AC)–specific TFEB knockout mice develop severe pancreatitis after Gao-binge alcohol.** BAC-Ela-Cre-; TFEB f/f (TFEB WT) and BAC-Ela-Cre+; TFEB f/f (TFEB KO) mice were injected with tamoxifen (75 mg/kg) once a day for consecutive 5 days, then treated with the Gao-binge alcohol model. (*A*) Immunofluorescence staining of LAMP1 was performed using cryosections of the mouse pancreas followed by confocal microscopy. Data are mean ± SE (n = 4). **P < .01; Student’s *t*-test analysis. Scale bar = 20 μm. (*B*) Quantification of autophagic vacuoles by EM from TFEB WT and TFEB KO mice fed with liquid control diet. More than 20 fields from each group were counted. *P < .05; Student’s *t*-test analysis. (*C*) Trypsin activity was measured from alcohol-fed mouse pancreatic tissues. Data are mean ± SE (n = 6–8). **P < .01; Student’s *t*-test analysis. (*D*) Representative images of hematoxylin and eosin staining are shown. Arrowheads denote infiltrated inflammatory cells, omega shows pancreatic edema, asterisk indicates ADM, and delta represents cell death. (*E*) Individual histology score of hematoxylin and eosin staining was graded and data are mean ± SE (n = 5). **P < .01; Student’s *t*-test analysis. (*F*) Representative images of Ly6B, CK19, and Sirius red staining are shown. (*G*) Representative EM images of TFEB WT and TFEB KO mice after alcohol are shown. Arrows denote enwrapped ZGs within autophagic vacuoles. Scale bar = 500 nm for TFEB WT EtOH and 2 μm for TFEB KO EtOH. AL, autolysosome; AP, autophagosome; L, lysosome; NEU, neutrophil.

**Figure 8:**
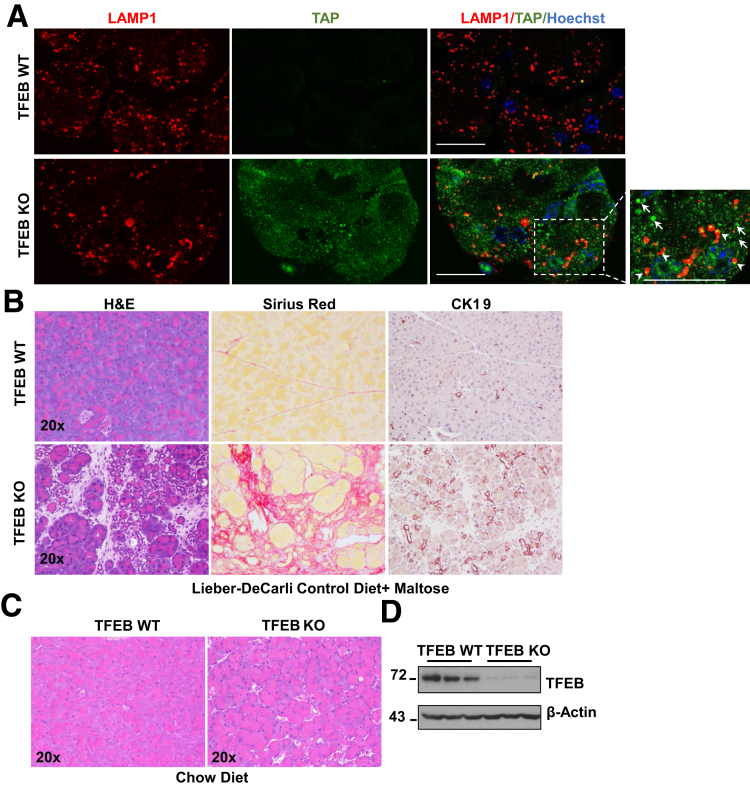
**Acinar cell–specific TFEB KO mice develop severe pancreatitis with the Lieber-DeCarli liquid diet but not with the chow diet.** BAC-Ela-Cre-; TFEB f/f (TFEB WT) and BAC-Ela-Cre+; TFEB f/f (TFEB KO) mice were injected with tamoxifen (75 mg/kg) once a day for consecutive 5 days, then fed with the liquid control diet or chow diet. (*A*) Representative immunofluorescence images of LAMP1 (red) and TAP (green) from TFEB WT and TFEB KO mice fed with the liquid control diet. Boxed area was enlarged and shown on the right. The arrowhead shows colocalized puncta of LAMP1 and TAP; the arrow denotes TAP-positive puncta. Scale bar = 20 μm. (*B*) Representative hematoxylin and eosin (H&E), Sirius red, and CK19 staining images of liquid control diet–fed mouse pancreas are shown. (*C*) H&E staining images of chow diet–fed mouse pancreas are shown. (*D*) Total pancreatic lysates from chow diet–fed TFEB WT mice and TFEB KO mice were subjected to Western blot analysis.

To determine whether decreased acinar cell TFEB would play a causal role in alcohol-induced pancreatitis, we generated inducible acinar cell–specific TFEB knockout (KO) mice and subjected these mice and their matched wild-type (WT) mice to Gao-binge alcohol model. We found that the number of LAMP1-positive puncta decreased almost 60% in the TFEB KO pancreas compared with TFEB^f/f^ (WT) mice, suggesting decreased lysosome numbers in the TFEB KO pancreas (Figure 7*A*). Decreased lysosome numbers in TFEB KO mouse acinar cells were also found by EM analysis (Figure 7*B*). Furthermore, alcohol-fed TFEB KO mice showed markedly elevation of trypsin activity compared with alcohol-fed TFEB WT mice (Figure 7*C*). Alcohol-fed TFEB KO mice had decreased numbers of acinar cells but increased acinar cell death, acinar-to-ductal metaplasia (ADM), and infiltration of inflammatory cells (Figure 7*D*, *E*). In addition, TFEB KO mice had increased Ly6B, cytokeratin 19 (CK19), and Sirius Red staining compared with TFEB WT mice (Figure 7*F*). EM studies further revealed that acinar cell–specific TFEB KO mice had decreased numbers of acinar cells and ZGs but increased numbers of pancreatic stellate cells, infiltrated inflammatory cells (neutrophils), and ADM (Figure 7*G*). Intra-acinar cell activation of trypsinogen likely occurs in autolysosomal compartments, which has been implicated in the pathogenesis of pancreatitis.^29^^,^^30^ To determine the cellular location of trypsinogen activation in TFEB KO acinar cells, we performed the immunostaining using the trypsinogen activation peptide antibody for activated trypsinogen (TAP) and LAMP1 for autolysosome or lysosome compartments. We found that some of the TAP staining puncta indeed colocalized with LAMP1 compartments, whereas some TAP puncta did not colocalize with LAMP1-positive compartments (Figure 8*A*). These data suggest that trypsinogen can be activated outside of autolysosomes or lysosomes in addition to autolysosome or lysosome compartments. To our surprise, the Lieber-DeCarli liquid control diet–fed TFEB KO mice also had increased edema, ADM, infiltration of inflammatory cells, and cell death, as well as elevated Sirius Red and CK19 staining (Figure 8*B*). In contrast, TFEB KO mice fed with regular chow diet only had mild pancreatic histological changes (Figure 8*C*). Immunoblotting analysis showed markedly decreased levels of TFEB in TFEB KO mice, indicating successful deletion of TFEB in mouse pancreas (Figure 8*D*). These data suggest that genetic deletion of TFEB in mouse acinar cells alone is not sufficient to trigger noticeable pancreatitis at the basal conditions. However, deletion of TFEB in acinar cells markedly exacerbates pancreatic degeneration, inflammation, and fibrosis, which mimic the features of chronic pancreatitis, after the mice were fed with Lieber-DeCarli liquid diet regardless of ethanol feeding.

### Acinar Cell–Specific Atg5 KO Mice Develop Severe Pancreatitis With the Lieber-DeCarli Liquid Diet Regardless of Ethanol

**Figure 9:**
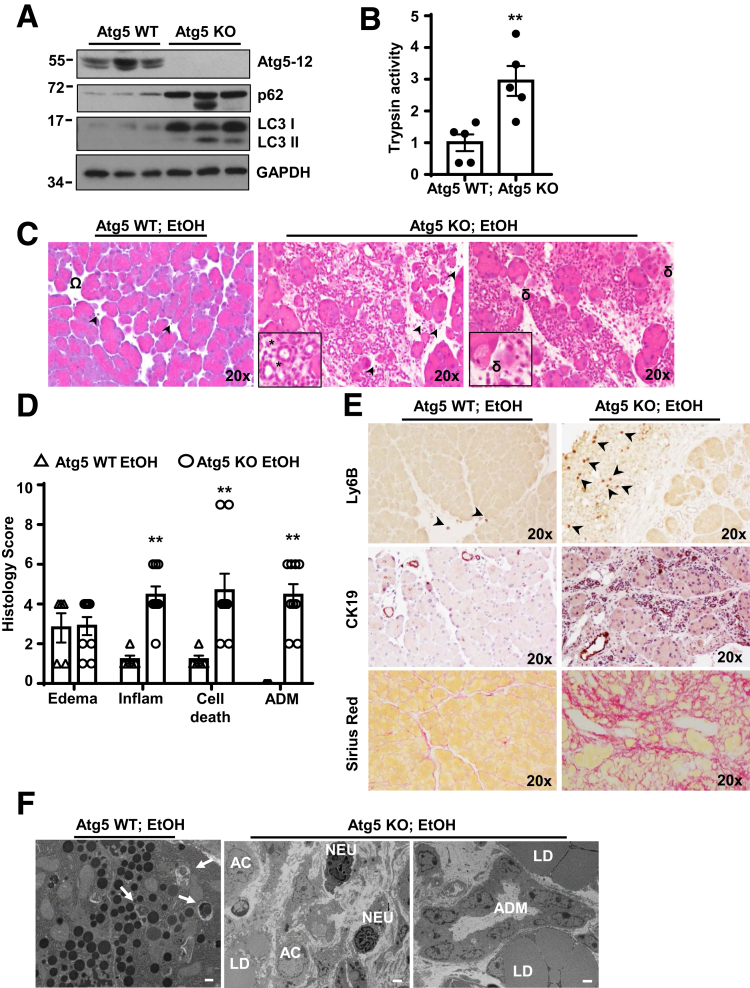
**Acinar cell (AC)–specific Atg5 knockout mice develop pancreatitis regardless of alcohol feeding.** BAC-Ela-Cre-; Atg5 f/f (Atg5 WT) and BAC-Ela-Cre+; Atg5 f/f (Atg5 KO) mice were injected with tamoxifen (75 mg/kg) once a day for consecutive 5 days, then treated with the Gao-binge alcohol model. (*A*) Total pancreatic lysates were subjected to immunoblot analysis. (*B*) Trypsin activity was measured from ethanol-treated pancreatic tissues. Data are mean ± SE (n = 5). **P < .01; Student’s *t*-test analysis. (*C*) Representative images of hematoxylin and eosin staining are shown. Arrowheads denote infiltrated inflammatory cells, omega shows pancreatic edema, asterisk indicates ADM), and delta represents cell death. Boxed areas were enlarged and showed in the lower panel. (*D*) Individual histology score of hematoxylin and eosin staining was graded and data are mean ± SE (n = 5–9). **P < .01; Student’s *t*-test analysis. (*E*) Representative images of Ly6B, CK19, and Sirius red staining are shown. (*F*) Representative EM images of Atg5 WT and Atg5 KO mice after alcohol are shown. Arrows denote enwrapped ZGs within autophagic vacuoles. LD, lipid droplet; NEU, neutrophil. Scale bar = 500 nm for Atg5 WT EtOH and 2 μm for Atg5 KO EtOH.

TFEB-mediated lysosomal biogenesis mainly acts at the late stage of autophagy, we next wondered whether blocking the upstream autophagosome formation would also affect alcohol-induced pancreatitis. We thus generated inducible acinar cell–specific Atg5 KO mice and subjected these mice to Gao-binge alcohol model. Immunoblotting analysis revealed that the levels of conjugated Atg5-12 markedly decreased accompanied with increased LC3-I and p62 levels in Atg5 KO mice compared with Atg5 WT mice, suggesting that autophagy was efficiently blocked in the acinar cell–specific Atg5 KO mouse pancreas (Figure 9*A*). Alcohol-fed Atg5 KO mice showed marked elevation of trypsin activity compared with alcohol-fed Atg5 WT mice (Figure 9*B*). Similar to acinar cell–specific TFEB KO mice, alcohol-fed Atg5 KO mice had decreased numbers of acinar cells but increased numbers of acinar cell death, increased ADM, infiltration of inflammatory cells, and CK19 staining, as well as increased Sirius Red staining, compared with Atg5 WT mice (Figure 9*C–E*). EM studies confirmed that acinar cell–specific Atg5 KO mice had decreased numbers of acinar cells and ZGs but had increased numbers of inflammatory cells (neutrophils) and large lipid droplets, as well as increased ADM (Figure 9*F*). Intriguingly, similar to TFEB KO mice, the Lieber-DeCarli liquid control diet–fed Atg5 KO mice had markedly increased ADM, infiltration of inflammatory cells and cell death as well as elevated Sirius Red and CK19 staining (data not shown). Acinar cell–specific Atg5 KO mice fed with regular chow diet only showed mild edema and infiltration of inflammatory cells (data not shown), which is consistent with a previous report.^7^ Together, these data indicate that genetic deletion of Atg5 in acinar cells promotes the fibrotic pancreatitis after the mice were fed with the Lieber-Decarli liquid diet regardless of ethanol feeding, which is similar to TFEB KO mice.

### Overexpression of TFEB Ameliorates Alcohol-Induced Pancreas Injury

**Figure 10:**
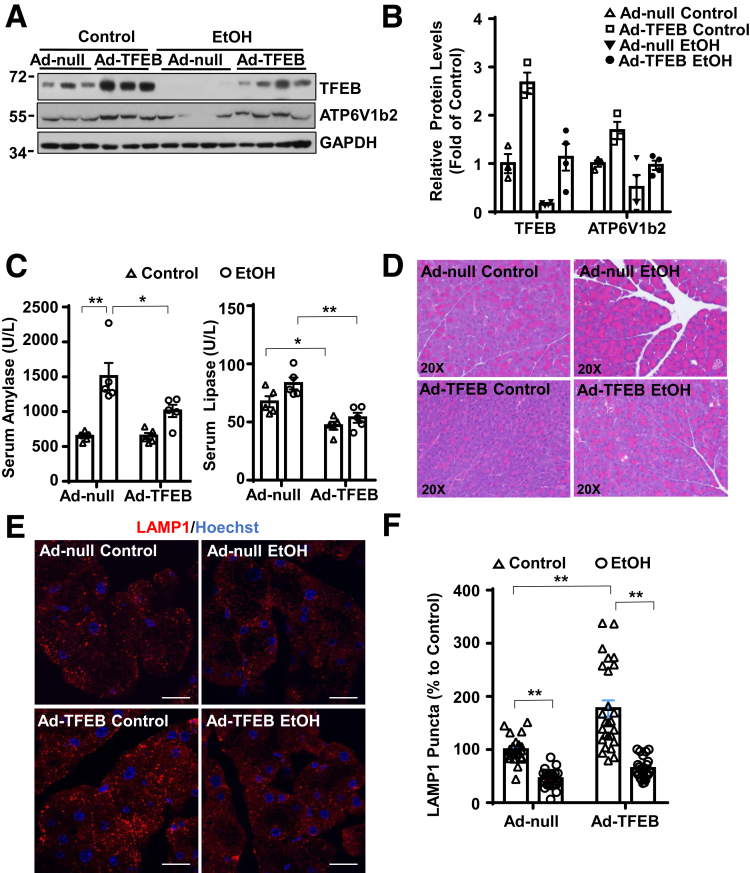
**Overexpression of TFEB protects against Gao-binge alcohol-induced pancreatitis in mice.** (*A*) WT C57Bl/6J mice were injected with Ad-Null and Ad-TFEB (5 × 10^8^ PFU/mouse via tail vein) followed by the Gao-binge alcohol model. Total mouse pancreatic lysates were subjected to immunoblot analysis. (*B*) Densitometry analysis of panel *A*. Data are mean ± SE (n = 3–4). (*C*) Serum amylase and lipase activities were measured after the Gao-binge alcohol model. Data shown are mean ± SE (n = 4–6). *P < .05; **P < .01 by 1-way analysis of variance. (*D*) Representative hematoxylin and eosin images are shown. (*E*) Immunofluorescence staining of LAMP1 using cryosections from Ad-Null and Ad-TFEB pancreas tissues. Scale bar = 20 μm. (*F*) Quantification of LAMP1 puncta from panel *E*. Data are mean ± SE (n = 3–4) from at least 22 images in each group. **P < .01 by 1-way analysis of variance.

To investigate whether TFEB could protect against ethanol-induced pancreas injury, we overexpressed TFEB through tail vein injection of adenovirus-TFEB (Ad-TFEB). We found that administration of Ad-TFEB reversed alcohol-induced decrease of TFEB and its target protein ATP6V1b2 in the mouse pancreas (Figure 10*A*, *B*). In addition, overexpression of TFEB significantly attenuated alcohol-induced pancreatic injury, as demonstrated by decreased levels of serum amylase and lipase as well as decreased pancreatic edema and ZG accumulation (Figure 10*C*, *D*). Moreover, overexpression of TFEB increased LAMP1 staining in the control group and partially reversed the reduction of LAMP1 induced by alcohol (Figure 10*E*, *F*). These data indicate that overexpression of TFEB protects against alcohol-induced pancreatitis.

### Evidence for Impaired TFEB-Mediated Lysosomal Biogenesis in Human Alcoholic Pancreatitis

**Figure 11:**
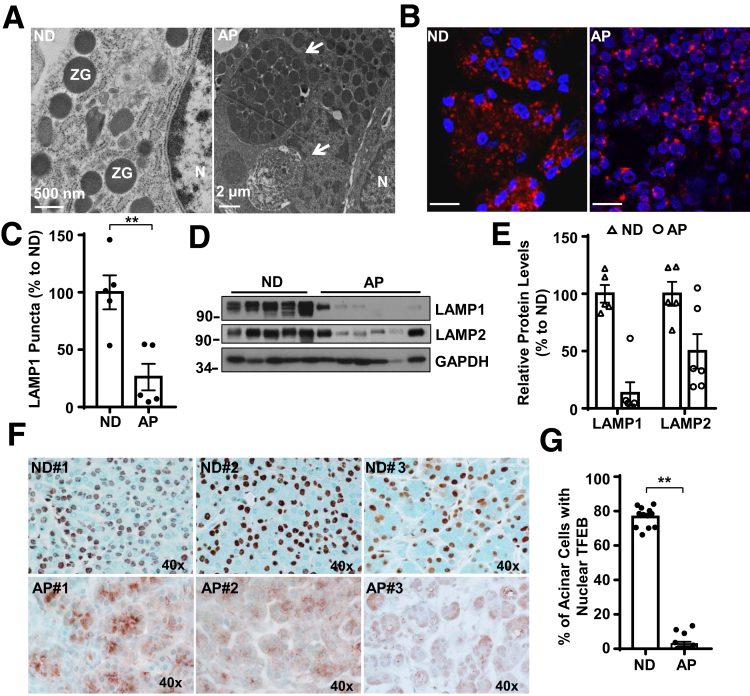
**The levels of nuclear TFEB and pancreatic LAMP1 decrease in human pancreatitis.** (*A*) Representative EM images of acinar cells from human normal donor (ND) and alcoholic pancreatitis (AP) pancreas are shown. Arrows denote large autolysosome. Scale bar = 500 nm for ND and 2 μm for AP. (*B*) Immunofluorescence analysis of LAMP1 staining using cryopancreatic tissues from ND and AP. Scale bar = 20 μm. (*C*) LAMP1-positive puncta per cell in each group were quantified (n = 5) from panel *B*. Data are mean ± SE. More than 60 cells were counted in each sample. (*D*) Immunoblotting analysis using total lysates from ND and AP pancreatic tissues. (*E*) Densitometry analysis of panel *D*. LAMP1 and LAMP2 levels were normalized to GAPDH of the same sample. (*F*) Representative images of TFEB immunohistochemistry staining in ND and AP tissues are shown. (*G*) The number of cells with nuclear TFEB staining were counted in each group (n = 12 for ND, n = 14 for AP). Data are mean ± SE, and at least 6 images were counted in each sample. **P < .01 by Student’s *t* test. N, nucleus.

To determine whether impaired TFEB-mediated lysosomal biogenesis would be associated with human pancreatitis, we first performed EM studies on pancreatic tissues from normal healthy donors and alcoholic pancreatitis patients that we obtained from the Liver Center of University of Kansas Medical Center. Consistent with a previous report,^31^ large intracellular autolysosomal vacuoles containing enwrapped ZGs or degraded contents readily detected in pancreatic acinar cells of alcoholic pancreatitis patients but not in normal healthy control pancreatic tissues (Figure 11*A*). Immunofluorescence staining showed punctate LAMP1 staining in acinar cells in both normal and alcoholic pancreatitis samples (Figure 11*B*), but the number of LAMP1 puncta decreased significantly in alcoholic pancreatitis pancreas (Figure 11*C*). Furthermore, immunoblotting analysis also revealed decreased levels of pancreatic LAMP1 and LAMP2 proteins in alcoholic pancreatitis patients compared with normal healthy control donors (Figure 11*D*, *E*). These data suggest that human pancreatitis may have decreased numbers of lysosomes in acinar cells. Hematoxylin and eosin staining revealed increased infiltration of inflammatory cells, ductal structures, and fibrosis areas in pancreatic tissues from alcoholic pancreatitis patients (data not shown), confirming the typical features of chronic pancreatitis. Immunohistochemistry and immunofluorescence staining of TFEB in pancreas revealed that TFEB mainly localized in the nucleus of acinar cells in normal human pancreatic tissues, but TFEB largely displayed a cytosolic pattern in acinar cells from human alcoholic pancreatitis tissues (Figure 11*F*, *G* and data not shown). These data indicate that alcoholic human pancreatitis is associated with impaired TFEB-mediated lysosomal biogenesis, which is similar to Gao-binge alcohol-induced pancreatitis in mice.

## Discussion

In the present study, we found that chronic feeding plus acute binge of alcohol induced typical features of pancreatitis with pancreatic edema, increased ZG accumulation, inflammation, and increased serum levels of amylase and lipase. Mechanistically, alcohol increased autophagosome formation but decreased TFEB protein, resulting in impaired lysosomal biogenesis in mouse pancreas. Consequently, alcohol decreased lysosome numbers leading to insufficient autophagy to remove fragile ZGs in mouse pancreas. Pancreatic acinar cell–specific depletion of ATG5 or TFEB in the mouse developed fibrotic pancreatitis regardless of alcohol feeding. In contrast, overexpression of TFEB rescued alcohol-induced pancreatic injury. More importantly, decreased nuclear TFEB and lysosome numbers were also observed in human alcoholic pancreatitis samples.

Alcohol abuse plays a critical role in the development of chronic pancreatitis.^32^ However, only a small portion of people who abuse alcohol eventually develop pancreatitis.^14^ Chronic alcohol feeding causes mild pancreatic damages in rodents but can promote more severe pancreatitis in the presence of endotoxin, smoking, or cholecystokinin.^13^^,^^33^, ^34^, ^35^, ^36^ Although genetic factors such as mutations of cationic trypsinogen gene (PRSS1) are strongly associated with pancreatitis,^37^ acinar cells may develop adaptive response to protect against insults from alcohol abuse and other environmental factors. These genetic factors together with the cellular adaptive response may explain why only a small portion of alcohol abusers develop severe pancreatitis. Once the adaptive responses are compromised either genetically or pharmacologically, severe pancreatitis may occur. Among the adaptive responses, autophagy plays a critical role to maintain the homeostasis of acinar cell function, as the whole pancreas deletion of Atg5 led to the development of spontaneous pancreatitis.^7^ Lysosomes sit at the last step of autophagy by fusing with autophagosomes to form autolysosomes for degradation of contents transported from autophagosomes. Interestingly, mice with defective lysosomal function by genetic deletion of LAMP2 also developed spontaneous pancreatitis.^8^ These observations indicate that impaired autophagy-lysosomal pathway may promote pancreatitis. Here, we provide novel evidence that alcohol impaired TFEB that led to decreased lysosomal biogenesis and insufficient autophagy in the mouse pancreas. It is likely that impaired TFEB-mediated lysosomal biogenesis by alcohol consumption may prime these individuals to be more prone to develop pancreatitis in the presence of other insults such as smoking.

TFEB is a basic helix-loop-helix leucine zipper transcription factor that directly binds to the CLEAR motif found within the promoters of lysosomal genes. By binding with the CLEAR motif, TFEB can upregulate the entire CLEAR network of genes involved in lysosomal biogenesis and lysosomal functions.^17^^,^^18^ The activity and cellular distribution of TFEB are tightly controlled by its posttranslational level through phosphorylation of specific amino acid residues. To date, at least 6 kinases, including mTOR, MAPK1, GSK3β, AKT, MAP4K3, and PKCβ have been identified to regulate TFEB function by phosphorylation.^38^^,^^39^ mTOR phosphorylates TFEB at S122, S142, and S211; MAPK1 at S142; GSK3β at S134 and S138; AKT at S467; and MAP4K3 at S3, which lead to cytoplasmic retention and proteasome degradation of TFEB. In contrast, PKCβ phosphorylates TFEB at S462, S463, S467, and S469 stabilizes and activates TFEB.^20^^,^^39^, ^40^, ^41^ One of the intriguing findings in this study was that pancreatic TFEB protein was markedly decreased after alcohol treatment. We previously reported that chronic feeding plus binge alcohol activated mTOR but not MAPK1 in mouse livers, which led to decreased TFEB proteins in mouse livers.^22^ In contrast, we found that alcohol inhibited mTOR but increased MAPK1 activation in the mouse pancreas. At present it is unclear why alcohol activated different kinases in different tissues for TFEB phosphorylation and subsequent degradation. Decreased pancreatic TFEB proteins after alcohol could be due to 2 possible mechanisms: transcription level and posttranslational degradation. The decreased messenger RNA levels of pancreatic TFEB after alcohol may support a role of transcription regulation. The slightly increased pancreatic proteasome activity after alcohol may also suggest a possible role of proteasomal degradation of TFEB. Future studies are needed to further characterize the differential contributions of transcription vs degradation on alcohol-induced decrease of TFEB and pancreatic pathogenesis.

Perhaps the most intriguing findings in this study are the acinar cell–specific Atg5 or TFEB KO mice developed severe pancreatic changes reminiscent of chronic pancreatitis in the Lieber-DeCarli control diet–fed mice regardless of alcohol feeding. Because the acinar cell–specific Atg5 or TFEB KO mice only had mild pancreatic histological changes when these mice were fed with a regular chow diet, it is likely that either the administration route (liquid vs chow) or some of the unidentified nutrient factors in the Lieber-DeCarli diet triggered the pancreatic damage. While a high-fat diet has been associated to pancreatitis, a Western diet that contains more than 40% fat did not cause more injury in these acinar cell–specific Atg5 or TFEB KO mice (data not shown). While more studies are definitely needed to identify the causal factors in this Lieber-DeCarli diet feeding model, our observations clearly support the notion that lack of TFEB-mediated lysosome biogenesis and autophagy in mice will prime these mice to be more sensitive to stresses to develop pancreatitis.

How does autophagy-lysosomal pathway protect against the pathogenesis of pancreatitis? Impaired lysosome functions have been implicated in human pancreatitis, and accumulation of ZGs and autophagosomes are readily seen in pancreatitis tissues.^8^^,^^31^^,^^42^ In this study, we found increased colocalization of GFP-LC3–positive autophagosomes and LAMP1-positive lysosomes or autolysosomes with amylase-positive ZGs as well as enveloped ZGs in autophagy vacuoles by EM, supporting a possible role of autophagy-lysosome in removal of ZGs. These findings support the hypothesis that autophagy-lysosome may protect the acinar cells at least by removing the fragile damaged ZGs, which otherwise may cause intracellular leakage and trypsin activation that leads to pancreatitis. While we found autophagosome marker LC3-II was enriched in the isolated ZGs after Gao-binge alcohol, we could not rule out that ZG fractions might be contaminated with autophagosomes. Future work is needed to isolate autophagosomes from pancreatitis tissues to determine whether the isolated autophagosomes would also contain ZGs. Nevertheless, we also found abnormally large vacuoles containing undegraded ZGs or partially degraded contents in human alcoholic pancreatitis samples. Decreased lysosome and nuclear TFEB staining were also observed in these pancreatitis samples. Thus, our results also imply that the impaired-TFEB-mediated lysosomal biogenesis not only contributes to the experimental alcoholic pancreatitis but also to human alcoholic pancreatitis.

In conclusion, our data demonstrate that the TFEB-mediated lysosomal biogenesis and autophagy pathway serve as critical adaptive mechanisms to protect the pancreatic acinar cells against alcohol-induced injury by maintaining the homeostasis of pancreas. Hence, targeting TFEB-mediated lysosomal biogenesis may be a promising therapeutic approach to prevent and treat pancreatitis.

## Materials and Methods

### Animal Model of Pancreatitis

Alcoholic acute pancreatitis was induced in C57Bl/6J and GFP-LC3 transgenic mice using the recently established chronic feeding with acute binge (Gao-binge alcohol) mouse model.^24^ GFP-LC3 mice were generated as described previously^43^ and C57Bl/6J mice were obtained from The Jackson Laboratory. Briefly, male mice (8 to 12 weeks old) were acclimated to the Lieber-DeCarli liquid diet (F1259SP; Bio-Serv, Flemington, NJ) for 5 days followed by ethanol (Decon)-containing (5%, v/v) liquid diet (F1258SP; Bio-Serv) supplemented with maltose dextrin (Bio-Serv) for 10 days. In the morning of the last day, the mice were orally gavaged with ethanol (5 g/kg) or calorie- and volume-matched maltose dextrin. For autophagic flux experiment, 1 dose of leupeptin (40 mg/kg, intraperitoneal) was given right before the gavage on the final day. For TFEB overexpression experiment, 1 dose of Ad-TFEB (5 × 10^8^ PFU/mouse, intravenous) or Ad-Null (5 × 10^8^ PFU/mouse, intravenous) was given on the first day of ethanol feeding. Mice were euthanized 8 hours after the gavage, and blood samples and pancreatic tissues were harvested. Pancreas injury was determined by measuring serum amylase and lipase activities. All procedures were approved by the Institutional Animal Care and Use Committee of the University of Kansas Medical Center.

### Generation of Pancreatic Acinar Cell–Specific Atg5 or TFEB KO Mice

Atg5 flox/flox and TFEB flox/flox mice were generated as described previously^41^^,^^44^ and crossed with BAC-Ela-CreErT transgenic mice (The Jackson Laboratory, Bar Harbor, ME) to generate tamoxifen-inducible pancreatic acinar cell–specific Atg5 or TFEB KO mice. Tamoxifen-inducible Cre-ErT was activated by intraperitoneal injection of 75 mg/kg tamoxifen (T5648; Sigma-Aldrich, St. Louis, MO) to 8-week-old mice for 5 days. Cre-negative littermates also received same tamoxifen treatment as previously described and served as control animals. Mice were further treated with the Gao-binge alcohol model.

### Human Samples

Consent, corresponding case reports, 12 healthy human donors and 14 pancreatitis samples with alcohol consumption history were facilitated and provided by the University of Kansas Medical Center Liver Center in a de-identified manner with an institutional review board–approved protocol.

### Antibodies

The antibodies used for this study were AMYLASE (A8273; Sigma-Aldrich), Atg5 (PM050, MBL, Woburn, MA), phos-TFEB (ABE1971; Millipore, Burlington, MA), TFEB (A303-673A; Bethyl Laboratories, Montgomery, TX), CK19 (TROMA-III, Developmental Studies Hybridoma Bank, Iowa City, IA), GAPDH (2118; Cell Signaling Technology, Danvers, MA), p62 (H00008878-M01; Abnova, Taipei, Taiwan), phos-4EBP1 (9451; Cell Signaling Technology), 4EBP1 (9452; Cell Signaling Technology), phos-MAPK (9101; Cell Signaling Technology), MAPK (9102; Cell Signaling Technology), phos-S6-ribosomal protein (4858; Cell Signaling Technology), S6-ribosomal protein (2217; Cell Signaling Technology), GFP (sc-9996; Santa Cruz Biotechnology, Dallas, TX), LAMIN A/C (2032; Cell Signaling Technology), LAMP1 (1D4B and H4A3; Developmental Studies Hybridoma Bank, Iowa City, IA), LAMP2 (ABL-93 and H4A4; Developmental Studies Hybridoma Bank), and PGC1α (PAB12061; Abnova). The TAP antibody was generated by Thomas Kolodecik from Yale University.^45^ Antibodies for VATP6V1a and VATP6V1b2 are gifts from Dr. Dennis Brown from Harvard Medical School. The Anti-LC3 antibody was generated as previously described.^43^ HRP-conjugated, FITC-conjugated and Rhodamine-conjugated secondary antibodies were from Jackson ImmunoResearch (West Grove, PA).

### Isolation of ZGs

ZGs were prepared from WT C57Bl/6J mouse pancreas, as described previously.^46^ Briefly, after the acute-on-chronic alcohol feeding, 2 mouse pancreas were quickly excised and put into ice-cold homogenization buffer (250-mM sucrose, 5-mM MOPS, pH 7.0, 0.1 -mM PMSF, and 0.1-mM MgSO_4_). The fat and connective tissue were trimmed off and the pancreas was minced and suspended in 20-mL cold homogenization buffer. The suspension was homogenized for 15 seconds at the lowest speed with Bio-Gen PRO200 homogenizer (PRO Scientific, Oxford, CT), followed by 15 up-and down strokes with tight-fitting, glass-Teflon homogenizer. The homogenate was centrifuged at 450 g for 15 minutes to remove unbroken cells and nuclei. The supernatant was centrifuge twice at 1300 g for 15 minutes to get crude ZG pellets, which consist of mitochondria and ZGs. The crude ZG pellets were further mixed with Percoll (GE17-0891-01; Sigma-Aldrich) to get 50% Percoll, 250-mM sucrose, 50-mM MES (pH 5.5), 0.2-mM EGTA, 0.1-mM MgSO_4_, and 0.1-mM PMSF. The suspension was centrifuged at 40,000 rpm for 15 minutes in Beckman 70.1 Ti rotor (Beckman Coulter, Brea, CA). The ZGs formed a dense white band at the bottom of the tube.

### Preparation of Tissue Lysate, Cellular Fractionation, and Immunoblot Analysis

Frozen pancreatic tissues were sonicated on ice in radioimmunoprecipitation assay buffer supplemented with protease inhibitor cocktail. Lysates were centrifuged for 30 minutes at 12,500 *g* and supernatants were collected and stored at –80°C. Nuclear and cytoplasmic fractions of pancreas tissue were prepared by using a commercial kit (78835; Thermo Fisher Scientific, Waltham, MA). Protein (30 μg) was separated by 10%–12% sodium dodecyl sulfate polyacrylamide gel electrophoresis gel before transfer to a polyvinylidene fluoride membrane. Membranes were probed using appropriate primary and secondary antibodies and developed with SuperSignal West Pico chemiluminescent substrate (34080; Life Technologies, Carlsbad, CA).

### RNA Isolation and Real-Time Quantitative Polymerase Chain Reaction

RNA was isolated from mouse pancreas using TRIzol reagent (15596-026; Ambion, Austin, TX) and was reverse-transcribed into complementary DNA using RevertAid Reverse Transcriptase (EP0442; Fermentas, Waltham, MA). Quantitative polymerase chain reaction (qPCR) was performed using SYBR Green chemistry (1725124; Bio-Rad Laboratories, Hercules, CA). Primer sequences (5′ to 3′) for primers used in qPCR are: *18s* F: TAGAGGGACAAGTGGCGTTC; *18s* R: CGATGAGCCAGTCAGTGT; *Cd68* F: TGCGGCTCCCTGTGTGT; R: TCTTCCTCTGTTCCTTGGGCTAT; *Il-1β* F: GCCCATCCTCTGTGACTCAT; R: AGGCCACAGGTATTTTGTCG; *Il-6* F: ACAACCACGGCCTTCCCTACTT; R: CATTTCCACGATTTCCCAGAGA; *Tnfα* F: CGTCAGCCGATTTGCTATCT; R:

CGGACTCCGCAAAGTCTAAG; *Lamp1* F: AGCATACCGGTGTGTCAGTG; R: GTTGGGGAAGGTCCATCCTG; *Lamp2* F: TGCAGTGCAGATGAAGACAAC; R: GCTATGGGCACAAGGAAGTTG; *Ly6g* F: TGCGTTGCTCTGGAGATAGA; R: CAGAGTAGTGGGGCAGATGG; *Tfeb* F: CCAGAAGCGAGAGCTCACAGAT, R: TGTGATTGTCTTTCTTCTGCCG; *Pgc1α* F: ATGTGTCGCCTTCTTGCTCT, R: ATCTACTGCCTGGGGACCTT; *Vatp6v1d* F: GAGCACAGACTGGTCGAAA, R: AGCTGTCAGTTCCTTCGTGG; *Vatp6v1h* F: ATGAGTACCGGTTTGCCTGG, R: GACTGAATGCCAGGAGCCAT; Real-time qPCR results were normalized to 18s and expressed as fold over control group.

### Immunostaining and Confocal Microscopy

Immunostaining for amylase, LAMP1, LAMP2, TFEB, and V-ATP6V1A was performed on pancreatic tissue cryosections. Images were acquired using a Leica TSC SPE confocal microscope with a 63× objective (Leica, Mannheim, Germany). Nuclei were counterstained with Hoechst33342 (H3570; Thermo Fisher Scientific). To assess the intensities of colocalization of 2 proteins, ImageJ 1.47v (National Institutes of Health, Bethesda, MD) colocalization plug-in was used (https://imagej.nih.gov/ij/plugins/colocalization.html).

### Histology and Immunohistochemistry

Paraffin-embedded pancreas sections were stained with hematoxylin and eosin and immunostaining for Ly6B, CK19, and TFEB. Sirius red staining was conducted with Direct red 80 (365548; Sigma-Aldrich). Images were taken using a Nikon Eclipse Ni microscope (Nikon, Tokyo, Japan).

### Trypsin Activity

Trypsin activity was measured in pancreatic tissue homogenates by fluorimetric assay, as described previously.^47^ Briefly, pancreas tissues were homogenized using a tight Teflon glass homogenizer in ice-cold buffer containing 5-mM MES (pH 6.5), 1-mM MgSO_4_, and 250-mM sucrose. The homogenates were added to the assay buffer containing 50-mM Tris-HCl, pH 8.0, 150-mM NaCl, 1-mM CaCl2, and 0.1-mg/mL bovine serum albumin. The reaction was initiated by incubating with a specific trypsin substrate, Boc-Gln-Ala-Arg-AMC (Enzo Life Sciences, Farmingdale, NY), which is converted to a fluorescent product by trypsin. The product was excited at 380 nm and emitted at 440 nm using Tecan plate reader (Tecan, Männedorf, Switzerland) and was followed for 6–10 minutes. Data were expressed as fold of control.

### Cathepsin B Activity

Cathepsin B activity was determined using mouse pancreas lysates. The pancreas tissues were lysed in ice-cold buffer containing 50-mM Tris (pH 7.4), 130-mM NaCl, 10% glycerol, 0.5% NP-40, 0.5-mM EDTA, and 0.5-mM EGTA. After centrifugation, total protein was normalized and then 15-μg protein from each sample was added to assay buffer containing 10-mM HEPES-NaOH (pH 7.4), 220-mM mannitol, 68-mM sucrose, 2-mM NaCl, 2.5-mM KH_2_PO4, 0.5-mM EGTA, 2-mM MgCl_2_, 5-mM pyruvate, and 1-mM dithiothreitol. The enzyme activity was estimated by adding cathepsin B substrate Z-Arg-Arg-AMC (Calbiochem, San Diego, CA) and measured at 355/460 nm. Data were expressed as fold of control.

### Electron Microscopy

Tissues were fixed with 2% glutaraldehyde in 0.1-M phosphate buffer (pH 7.4), followed by 1% OsO_4_. After dehydration, thin sections were stained with uranyl acetate and lead citrate for observation under a JEM 1016CX electron microscope (JEOL, Tokyo, Japan). Images were acquired digitally.

### Statistical Analysis

All experimental data were expressed as mean ± SE and subjected to 1-way analysis of variance with Bonferroni post hoc test or Student’s *t* test where appropriate. *P* < .05 was considered significant. All authors had access to the study data and had reviewed and approved the final manuscript.
